# Aspectos biomédicos y epidemiológicos del accidente ofídico en el departamento del Cauca, Colombia, 2009-2018

**DOI:** 10.7705/biomedica.5853

**Published:** 2021-06-15

**Authors:** María José Sevilla-Sánchez, Santiago Ayerbe-González, Eliana Bolaños-Bolaños

**Affiliations:** 1 Grupo de Investigación en Ecología Evolutiva, Departamento de Biología, Facultad de Ciencias Exactas y Naturales, Universidad de Nariño, Pasto, Colombia Universidad de Nariño Universidad de Nariño Pasto Colombia; 2 Grupo de Investigaciones Herpetológicas y Toxinológicas, Centro de Investigaciones Biomédicas - Bioterio, Universidad del Cauca, Popayán, Colombia Universidad del Cauca Universidad del Cauca Popayán Colombia; 3 Secretaría de Salud del Departamento del Cauca, Popayán, Colombia Secretaría de Salud del Departamento del Cauca Popayán Colombia

**Keywords:** mordeduras de serpientes, enfermedades desatendidas, Bothrops, Colombia, Snake bites, neglected diseases, Bothrops, Colombia

## Abstract

**Introducción.:**

El ofidismo en Colombia es un problema de salud pública, lo cual se hace evidente al examinar los datos epidemiológicos a nivel latinoamericano, pues el país ocupa el tercer lugar en número de accidentes ofídicos después de Brasil y México.

**Objetivo.:**

Hacer un análisis retrospectivo de los casos de accidente ofídico ocurridos entre 2009 y 2018 en el departamento de Cauca, según los datos del Sistema de Vigilancia en Salud Pública (Sivigila) registrados en el Instituto Departamental de Salud del Cauca.

**Materiales y métodos.:**

Se recopiló e interpretó la información consignada en las fichas de notificación obligatoria de accidente ofídico en los 10 años de estudio. Se determinaron la incidencia y la frecuencia de accidentes según la distribución geográfica y los agentes causales, y se analizaron las variables socioeconómicas relacionadas.

**Resultados.:**

Se registraron 1.653 casos y una baja mortalidad. Los géneros *Bothrops* y *Bothriechis* causaron la mayoría (77,43 %) de los accidentes, seguidos por el género *Micrurus* (2,9 %). La mayoría de las notificaciones procedían del sur del departamento; las personas de sexo masculino y los agricultores fueron los más afectados, con mordeduras en las extremidades superiores principalmente. Las principales manifestaciones del envenenamiento fueron las hemorrágicas, más frecuentes que la necrosis y la infección. Aunque el criterio para utilizar la seroterapia no siempre fue el mejor, las complicaciones iatrogénicas no fueron frecuentes.

**Conclusiones.:**

Los municipios de El Tambo y Piamonte, las personas de sexo masculino y las áreas rurales, fueron las variables más afectadas por el ofidismo, principalmente el ofidismo botrópico. Las mayores incidencias se presentaron en la zona sur del Cauca, en las cuencas de los ríos Patía y Caquetá.

## Abstract (en)

**Introduction:**

Snakebite is a relevant public health problem in Colombia, which is evident in epidemiological data at the Latin American level, where it ranks third place in the number of snakebites, after México and Brazil.

**Objective:**

To perform a retrospective analysis of snakebite accidents in the department of Cauca between 2009 and 2018 based on the data from the Public Health Surveillance System of Colombia reported to the Health Institute of Cauca.

**Materials and methods:**

We compiled and interpreted the information from the compulsory snakebite notification forms for the study’s 10 years period. We determined snakebite incidence and frequency according to the geographical distribution and the causal agents, and we analyzed related socioeconomic variables as well.

**Results:**

The records accounted for 1,653 cases and low mortality. *Bothrops* and *Bothriechis* species (77.43%) caused most of the accidents followed by *Micrurus* (2.9%). Most of the notifications came from the south of the department, and males and farmers were the most affected with bites mainly in the upper extremities. During poisoning, hemorrhagic manifestations predominated over necrosis and infections; serotherapy criteria were not always adequately applied, however, iatrogenic complications were not frequent.

**Conclusions:**

El Tambo and Piamonte municipalities, being male, and rural areas were the variables most affected by ophidism, mainly bothropic ophidism. The highest incidences occurred in the southern part of Cauca, in the Patía and Caquetá river basins.

El envenenamiento por mordedura de serpiente es potencialmente mortal. Se estima que a nivel mundial cerca de 5,4 millones de personas al año sufren accidentes ofídicos y, cerca de 2,7 millones de ellas, presentan envenenamiento; se registran entre 81.000 y 138.000 muertes al año, y el triple de casos culmina con amputación o discapacidad permanente ^1^.

En Colombia, se reporta una incidencia de entre 6,2 y 20 accidentes por mordedura de serpientes por cada 100.000 habitantes, con un índice de mortalidad del 4 al 7,6 %, valores que varían según la densidad poblacional y la riqueza de serpientes de la región ^2^. El 31,2 % de los casos ocurre en la región occidental, el 23,8 % en la Costa Atlántica, el 18,9 % en la Orinoquia, el 18,2 % en la región centro-oriental y el 7,7 % en la Amazonia ^3^, cifras que han llevado a que, después de Brasil y México, hoy Colombia sea el tercer país con mayor número de casos en Latinoamérica y, el cuarto, si se considera la estimación del número de casos en Venezuela, sobre todo en sus áreas rurales ^2^^,^^4^. Dada su localización en el neotrópico, esta región se cataloga como la de mayor incidencia de ofidismo ^5^.

Las condiciones ambientales y geográficas de Colombia favorecen una importante biodiversidad ofídica, con alrededor de 319 especies ^6^, 17 a 40% de las cuales se consideran de importancia médica según las regiones del país ^5^^,^^7^. Los datos de los accidentes pueden diferir según la densidad de la población, la abundancia de las especies venenosas y su distribución geográfica. El grado de envenenamiento y la aparición de complicaciones varían según la distancia entre el sitio del evento y los centros asistenciales, la disponibilidad de suero antiofídico, el tiempo transcurrido entre el accidente y la consulta, así como la capacitación del personal médico, entre otros factores.

En Colombia, las familias Viperidae y Elapidae son las de mayor importancia clínica, con 19 y 30 especies, respectivamente ^6^^,^^8^. En orden de importancia, las especies asociadas con el ofidismo pertenecen a los géneros *Bothrops*, *Crotalus*, *Bothriechis*, *Porthidium*, *Lachesis* y *Bothrocophias* (Viperidae), y *Micrurus* (Elapidae) ^3^^,^^7^^,^^9^^-^^11^. Las familias Colubridae y Dipsadidae ^12^ también incluyen especies de importancia en salud pública, aunque algunas pertenecientes a los géneros *Clelia*, *Erythrolamprus*, *Leptodeira*, *Helicops*, *Oxybelis*, *Oxyrhopus*, *Philodryas* y *Thamnodynastes* son opistoglifas, pero su veneno es de baja a moderada toxicidad ^12^. Otras, aunque no son venenosas, ocasionan mordeduras relativamente frecuentes y eventualmente pueden causar infecciones en el sitio de la mordedura; en este grupo cabe mencionar las serpientes aglifas de los géneros *Atractus*, *Chironius*, *Dipsas*, *Imantodes*, *Lampropeltis*, *Sibon* y *Xenodon*^13^^,^^14^.

A nivel mundial, nacional y regional, los intentos por determinar una tasa real de accidentalidad se ven limitados debido al alarmante subreporte existente, lo que dificulta entender el impacto verdadero de los accidentes ofídicos en las poblaciones, e implica grandes limitaciones y retos para los investigadores ^14^. En Colombia, el subreporte se ha estimado en 44,5 %, es decir, una cifra de accidentes anuales superior a 8.000. En este sentido, Cauca se ha catalogado como uno de los departamentos con mayor subreporte en el país (75,5 %), lo que equivale a decir que los casos podrían superar los 550 anuales ^15^.

En la categoría de enfermedades tropicales desatendidas, Cauca se considera un departamento con baja incidencia de mordeduras de serpientes en los boletines epidemiológicos semanales del Sistema de Vigilancia en Salud Pública (Sivigila) del Instituto Nacional de Salud de los últimos años ^16^^-^^18^. No obstante, no es posible asegurar que esta enfermedad tropical desatendida de categoría A, catalogada así por la Organización Mundial de la Salud (OMS) en el 2017 ^19^, realmente tenga baja incidencia en el departamento. Si bien los casos reportados al Sivigila han aumentado, no todos los accidentes son atendidos en los centros hospitalarios e, incluso cuando así sucede, no siempre son reportados, por lo cual el acumulado anual puede ser mucho mayor.

En el Cauca, se han hecho estudios que permiten vislumbrar que el accidente ofídico era un problema incluso antes de que fuera de notificación obligatoria en el país. En 1977, se hizo el primer estudio sobre el envenenamiento por mordedura de serpientes en Cauca; en él se revisó la herpetología clínica y su clasificación ^20^. En 1979, se hizo el primer análisis retrospectivo del ofidismo, considerando aspectos clínicos y epidemiológicos, así como las complicaciones. En él se propusieron grados de envenenamientos con base en los 48 casos atendidos en el Hospital Universitario San José de Popayán entre 1972 y 1977 ^7^. En un estudio retrospectivo del año 2000, se analizó un periodo de cinco años (1993- 1997) de accidentes ofídicos, en el cual se evaluaron la epidemiología, la etiología, la clínica y las complicaciones de 66 casos atendidos en el Hospital Universitario San José y, además, el tratamiento médico empleado ^21^. El último estudio sobre ofidismo en el Cauca cubrió el periodo entre el 2000 y el 2008, y en él se evaluaron el impacto epidemiológico del accidente ofídico, las principales especies de serpientes asociadas y su comportamiento sociodemográfico, así como los aspectos biomédicos de la enfermedad ^22^.

A partir de los registros de las fichas de notificaciones sobre ofidismo del Instituto Nacional de Salud (INS: 100), de la Dirección Territorial del Departamento del Cauca y de la Secretaría Departamental de Salud, en el presente estudio se describe retrospectivamente el comportamiento de los casos por mordedura de serpiente durante una década (2009-2018) y se hacen recomendaciones para el tratamiento clínico.

## Materiales y métodos

Con las modificaciones pertinentes, se siguió la metodología general propuesta por Cuéllar-Gordo, *et al.*^23^, Sevilla-Sánchez, *et al.*^14^, y el Instituto Nacional de Salud de Colombia, para el análisis epidemiológico retrospectivo de los casos.

### Área de estudio

El departamento del Cauca está ubicado al suroeste de Colombia, entre los 0°57’ y 3°20’ de latitud norte y los 75°45’ y 78°11’ de longitud oeste. Limita al norte con el departamento del Valle del Cauca, al oriente, con los departamentos de Tolima, Huila y Caquetá, al occidente, con el océano Pacífico, y al sur, con los departamentos de Nariño y Putumayo ^24^. Tiene una extensión de 29.308 km^2^, es decir, aproximadamente el 2,5 % del territorio colombiano, con una topografía dividida en seis subregiones: norte, centro, Pacífico, oriente, sur y macizo, y una estructura determinada principalmente por la llanura del Pacífico, las cordilleras Occidental y Central, el altiplano de Popayán, el Macizo Colombiano, el valle del Patía y el sector de la cuenca del Amazonas, las cuales incluyen desde zonas frías, como el nevado del Huila (5.631 msnm) y los volcanes de Puracé y Sotará, hasta tierras bajas en la costa del Pacífico ^24^.

### Población y muestra

Se analizaron los casos de accidente ofídico notificados al Sivigila por los 42 municipios del departamento, en el periodo comprendido entre el 2009 y el 2018, y se compararon principalmente con estudios preliminares en el departamento para evaluar la evolución de la enfermedad en el tiempo. Se incluyeron todos los casos ocurridos en el departamento del Cauca que fueron confirmados por la entidad de salud como accidente ofídico, o sea, pacientes con signos y síntomas, o sin ellos, mordidos por una serpiente, identificada o sin identificar. Se excluyeron aquellos casos ocurridos en Cauca y atendidos en otros departamentos, pues no se pudo acceder a la información, así como los que tenían datos duplicados y los casos erróneos en los que figuraba un agente causal cuya distribución y cuadro clínico no correspondían a los de la especie reportada, así como los casos ocurridos en otros departamentos pero atendidos en el Cauca ^14^^,^^23^.

### Recolección y análisis de la información

Se hizo un análisis retrospectivo a partir de la recopilación e interpretación de la información básica y complementaria consignada en las fichas de notificación epidemiológica para accidente ofídico (código INS: 100) de la Dirección Territorial del Departamento del Cauca y la Secretaría Departamental de Salud, los boletines semanales e informes parciales y anuales disponibles en la página web del Sivigila ^25^, y los datos de accidentes ofídicos reportados o atendidos por los autores.

### Variables epidemiológicas

Se evaluaron las siguientes variables consignadas en las fichas de notificación según los protocolos de vigilancia en salud pública establecidos por el Instituto Nacional de Salud y el Ministerio de Salud ^26^^,^^27^:

*1. Condiciones sociodemográficas del paciente:* semana epidemiológica en la que ocurrió el accidente, edad y sexo del paciente, municipio de residencia, y zona y cobertura de seguridad social

*2. Notificación del accidente:* clasificación inicial del caso, hospitalización y condición final del paciente

*3. Caracterización del accidente:* actividad en el momento del accidente, localización de la mordedura, género, especie y nombre común de la serpiente, este último, en caso de que el género no haya sido registrado, e identificación adicional según la procedencia y el cuadro clínico observado

*4. Manifestaciones del accidente y sus complicaciones:* signos locales (marcas de dientes o colmillos, edema, eritema, sangrado, equimosis, flictenas, linfangitis); síntomas locales (dolor, parestesias); síntomas sistémicos (cefalea, mareo, malestar general, sangrado por mucosas, vías respiratorias, digestivas, y urogenitales por *Bothrops* spp., *Bothriechis schlegelii*, *Bothrocophias* spp., *Porthidium nasutum*); disartria, dislalia, compromiso de pares craneanos, paresias, parálisis, paro respiratorio, mialgias o pigmenturia por *Crotalus durissus cumanensis*, *Micrurus* spp.; combinación de signos de hematotoxicidad, trombosis localizada y arritmias cardíacas por *Lachesis* spp. y gravedad del accidente (sin envenenamiento, leve, moderado o grave)

*5. Atención del accidente:* uso de suero antiofídico, y tipo y dosis del suero empleado

### Identificación y zoogeografía de los agentes causales

Los agentes causales fueron identificados teniendo en cuenta su procedencia, el examen del ejemplar llevado por el paciente o los familiares, o a partir del registro fotográfico, las manifestaciones clínicas, el reconocimiento del animal en un póster, o el nombre y la descripción dada por el paciente o sus familiares ^5^. Los signos y síntomas se catalogaron como locales y sistémicos, el grado de envenenamiento se determinó siguiendo las directrices de Ayerbe, *et al.*, en las “Guías para el manejo de las urgencias toxicológicas” del Ministerio de Salud y Protección Social ^28^, así como las de Cañas, *et al.*^29^, y las de Ángel ^30^.

Para comprender la diversidad y la distribución de la fauna ofídica, se tuvo en cuenta la división del departamento de Cauca en cinco cuencas hidrográficas, a lo que se integró la información registrada en las fichas de notificación obligatoria (INS: 100) con la información de bases de datos como la *Global Biodiversity Information Facility* (GBIF) y el Sistema de Información sobre Biodiversidad de Colombia (SIBColombia), así como los datos publicados por el Museo de Historia Natural de la Universidad del Cauca y el conocimiento de los autores, con el objetivo de trazar una distribución potencial de los géneros y especies de interés clínico a nivel departamental ^31^^-^^33^.

### Clínica del accidente

Se establecieron los diferentes síndromes observados según los agentes causales, y el tipo de complicación y manifestación local y sistémica que presentaron los pacientes. Se evaluaron el tipo de atención inicial, la aplicación de primeros auxilios o prácticas no médicas, y la seroterapia utilizada según el grado de envenenamiento, lo que se correlacionó con la zoogeografía de las especies presentes en el departamento del Cauca. Asimismo, se determinó el tipo de accidente con base en la sintomatología y el agente causal del accidente ofídico ^28^^,^^34^. En esta sección, se incluyeron, además, recomendaciones generales para la correcta atención del accidente ofídico con base en la experiencia médica, biológica y epidemiológica.

### Elaboración de mapas

Para la construcción de los mapas de georreferenciación de géneros y especies de interés clínico, se revisaron los registros de serpientes consignados en la Colección Herpetológica del Museo de Historia Natural de la Universidad del Cauca ^33^, los ejemplares mantenidos en el Centro de Investigaciones Biomédicas - Bioterio de la Universidad del Cauca (CIBUC- Bioterio), los datos de las notificaciones recogidas por Santiago Ayerbe, y las bases de datos de la GBIF ^32^ y del SIBColombia ^31^. Los mapas se elaboraron con el programa arcGIS™, versión 10.1, utilizando las planchas de Colombia, y del departamento de Cauca y sus municipios disponibles en el Instituto Geográfico Agustín Codazzi ^35^.

### Análisis estadístico

En el análisis estadístico de los datos, se emplearon medidas de tendencia central para las variables cuantitativas, y frecuencias relativas y absolutas y porcentajes para las variables cualitativas en el programa Excel™. La incidencia por año se calculó determinando el cociente entre el número de casos y la población del periodo 2009 a 2018, según las proyecciones demográficas del censo del Departamento Administrativo Nacional de Estadística (DANE) del 2005 para el departamento del Cauca ^36^

### Consideraciones éticas

Se garantizó la confidencialidad de la información, según lo estipulado en la Ley 1273 del 2009 y la Ley 1266 del mismo año.

## Resultados

### Tipos de accidentes ofídicos

**Figura 1:**
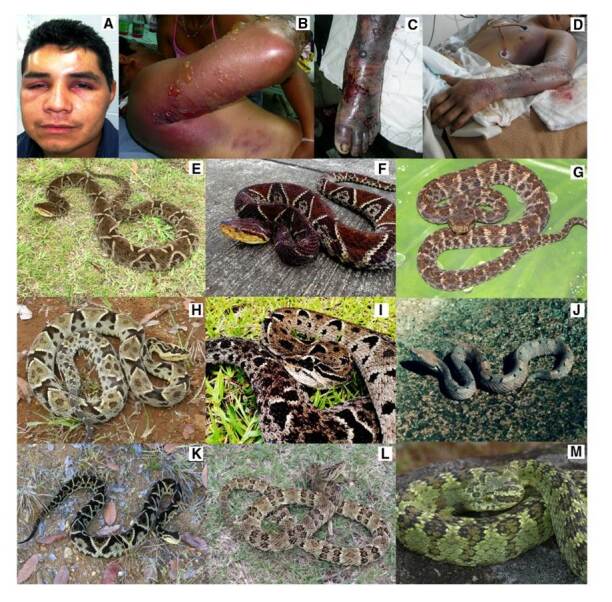
Manifestaciones clínicas y especies asociadas con ofidismo botrópico. **A.** Ofidismo grave por *Bothriechis schlegelii*. Mordedura en la cara. **B.** Ofidismo grave por *Bothrops rhombeatus.* **C.** Ofidismo grave por *Bothropsasper*. **D.** Gangrena por *Bothrops asper* (Cortesía del Hospital Universitario San José, Popayán). **E.** *Bothrops asper.* Terciopelo, equis negra. Localidad: corregimiento Huisitó, municipio El Tambo (cuenca del Pacífico), Cauca (Colombia). **F.** *Bothrops asper*. Localidad: municipio El Tambo (cuenca del Pacífico), Cauca (Colombia), **G.** *Bothrops atrox*. Cuatro narices, equis. Localidad: Piamonte, Cauca (Colombia), **H.** *Bothrops ayerbei*. Localidad: vereda Pomorroso, corregimiento San Joaquín, municipio El Tambo (cuenca del río Patía), Cauca (Colombia), **I.** Bothrops ayerbei. Localidad: municipio El Tambo (cuenca del río Patía), Cauca (Colombia), **J.** *Porthidium nasutum*. Hilván o patoquilla. Localidad: quebrada Guangüí, Timbiquí, Cauca (Colombia), **K.** *Bothrops rhombeatus*. Equis gata. Localidad: Cajibío, Cauca (Colombia), **L.** *Bothrops punctatus*. Equis rabo de chucha. Localidad: vereda Playa Rica, municipio El Tambo, Cauca (Colombia), **M.** *Bothriechis schlegelii.* Cabeza de candado, yaruma. Localidad: Cajibío, Cauca (Colombia).

**Figura 2:**
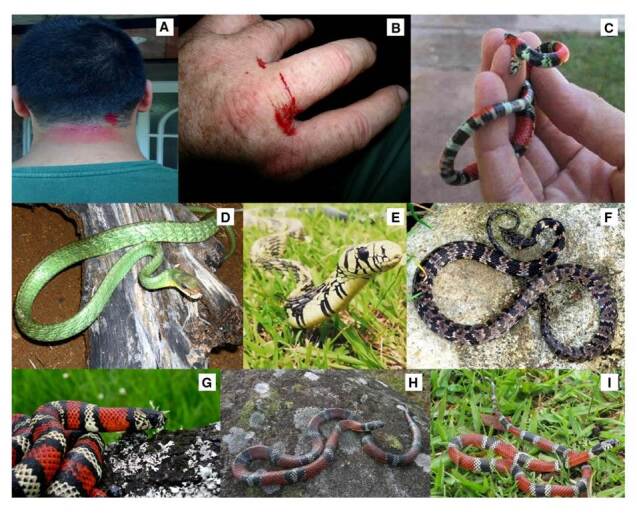
Manifestaciones clínicas y especies asociadas con ofidismo colúbrico. **A.** Ofidismo causado por *Spilotes pullatus* en Popayán **B.** Ofidismo causado por *Leptophis ahaetulla* (cortesía CEVAP/UNESP, Brasil). **C.** Ofidismo causado por *Leptophis ahaetulla*. **D.** *Chironius exoletus*. Guache, jueteadora. Localidad: vereda Pambío, municipio Timbío, Cauca (Colombia). **E.** *Spilotes pullatus*. Localidad: municipio Popayán, Cauca (Colombia) **F.** *Sibon nebulatus* popayanensis. Babosera, caracolera. Localidad: Popayán, Cauca (Colombia) **G.** *Lampropeltis micropholis*. Falsa coral. Localidad: Popayán, Cauca (Colombia) **H.** *Erythrolamprus bizona*. Falsa coral, mataganado. Localidad: Popayán, Cauca (Colombia). **I.** *Erythrolamprus pseudocorallus*. Falsa coral. Localidad: Inzá, Cauca (Colombia).

**Figura 3:**
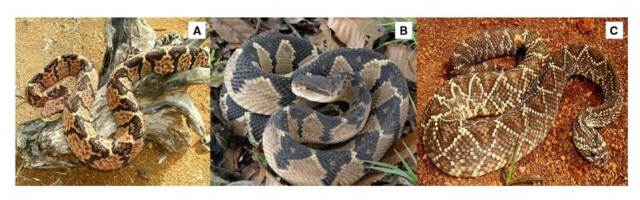
Especies asociadas con ofidismo lachésico y crotálico. **A.** *Lachesis acrochorda*. Verrugosa, guascama. Localidad: vereda Pocitos, corregimiento Huisitó, El Tambo, Cauca (Colombia). **B.** *Lachesis muta*. Rieca, martiguaja. Localidad: vereda Verdeyaco, corregimiento Santa Martha, municipio Santa Rosa, Cauca (Colombia). **C.** *Crotalus durissus cumanensis*. Cascabel. Localidad: municipio Garzón, departamento Huila (Colombia)

**Figura 4:**
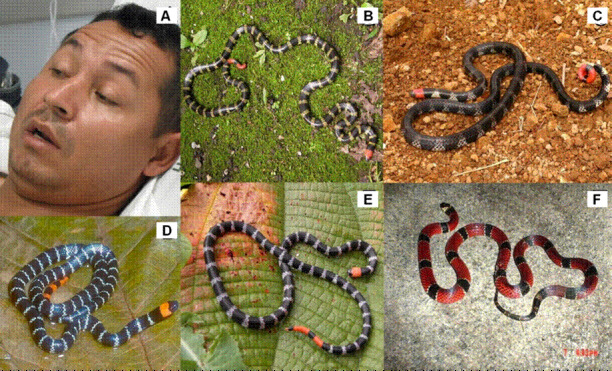
Manifestaciones clínicas y especies asociadas con ofidismo elapídico. **A.** Ofidismo causado por *Micrurus mipartitus decussatus*. **B.** *Micrurus mipartitus decussatus.* Rabo de ají andina. Localidad: Cajibío, Cauca (Colombia)*.* **C.** *Micrurus mipartitus popayanensis.* Rabo de ají payanesa. Localidad: Popayán, Cauca (Colombia) (cortesía CIBUC)*.* **D.** *Micrurus mipartitus anomalus.* Rabo de ají del Magdalena. Localidad: Huila (Colombia)*.* **E.** *Micrurus mipartitus mipartitus.* Rabo de ají del Pacífico. Localidad: Playa Rica, El Tambo, Cauca (Colombia). **F.** *Micrurus dumerilii transandinus.* coral verdadera. Localidad: vereda Pocitos, corregimiento Huisitó, municipio El Tambo, Cauca (Colombia)

Se determinaron los siguientes cinco tipos de accidentes ofídicos ocurridos en el departamento del Cauca entre el 2009 y el 2018, los cuales coinciden con los que generalmente se registran a nivel nacional y suramericano. No se encontraron reportes de accidentes boídicos o elapídico-pelámicos (*Hydrophis platurus)*. Así:

1. Accidente botrópico (*sensu lato*) (figura 1), asociado con el 77,43 % de los casos y con serpientes de los géneros *Bothriechis*, *Bothrops* y *Porthidium*, y las especies *Bothriechis schlegelii*, *Bothrops asper*, *B. atrox*, *B. ayerbei*, *B. punctatus*, *B. rhombeatus* y *Porthidium nasutum*

2. Accidente colúbrico (figura 2), asociado con el 2,12 % de los casos y con serpientes de las familias Colubridae y Dipsadidae

3. Accidente lachésico, asociado con el 0,60 % de los casos, y con las especies *Lachesis acrochorda* y *L*. *muta* (figura 3 A y B)

4. Accidente crotálico, asociado con el 0,18 % de los casos y con la víbora cascabel *Crotalus durissus cumanensis* (figura 3 C).

5. Accidente elapídico (micrúrico) (figura 4), asociado con el 2,9 % de los accidentes y principalmente con las especies *Micrurus mipartitus* y *M. dumerilii*.

Fotografías. A, B, C, D, E, G, H, J, K, L y M: Santiago Ayerbe-González; F e I: María José Sevilla-Sánchez (cortesía CIBUC)

Fotografías. A: Juan Camilo Viveros B; C, D, F y H: Santiago Ayerbe-González; E: María José Sevilla-Sánchez; G: Fernando Ayerbe-Quiñones (Cortesía CIBUC); I: Edwin Royer Coral

Fotografía: Santiago Ayerbe-González (cortesía de CIBUC)

Fotografías. A: Carlos Alberto Ordóñez-Delgado (cortesía de Clínica Valle del Lili, Santiago de Cali); B y E: Santiago Ayerbe-González; C: Fernando Ayerbe-Quiñones; D: Juan Manuel Renjifo- Rey; F: Jaime Ramírez

### Frecuencia e incidencia de los accidentes ofídicos

**Figura 5:**
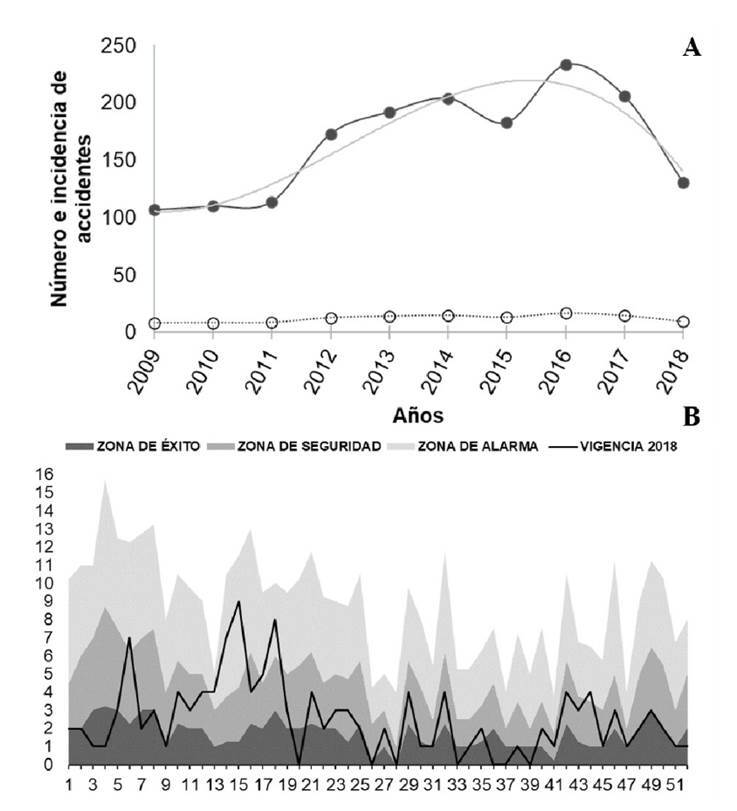
A. Número de casos e incidencia anual del accidente ofídico en el departamento del Cauca durante el periodo 2009-2018. La línea negra representa la tendencia del ofidismo en el departamento y, la línea gris, la incidencia. B. Canal endémico. Representación gráfica de las frecuencias del accidente ofídico a través del tiempo, incluidos casos máximos y mínimos notificados desde la semana 1 hasta la semana 52 durante el periodo 2009-2018, lo que permite determinar tendencias estacionales y marcar un antecedente histórico. El área gris oscura representa la zona de éxito. El área gris señala la zona de seguridad. El área gris clara señala la zona de alarma. La línea negra representa la vigencia para el 2018

Se registraron 1.653 accidentes ofídicos durante el periodo de 10 años, con un promedio de 165,3 casos por año, una incidencia anual de 12,1 casos por cada 100.000 habitantes y cinco defunciones registradas en los municipios de Argelia, El Tambo, López de Micay y Mercaderes, para una tasa de mortalidad del 0,3 %. Con base en los registros del Sivigila en el departamento, la incidencia anual durante los años analizados tuvo un incremento durante los primeros ocho años, pasando de 107 casos en el 2009 a 238 casos en el 2016, en tanto que, en el 2017 y el 2018, se observó un descenso en el número de accidentes, de 207 a 135. De todas maneras, fue clara una tendencia creciente en el número de casos atendidos (figura 5 A).

En cuanto al canal endémico, se pudo evidenciar que durante la vigencia del 2018, los reportes de las semanas epidemiológicas 6, 13, 14, 15, 17, 18 y 44 evidenciaron una zona de riesgo, especialmente la semana 15, en tanto que las semanas 3, 4, 20, 33, 36, 37 y 39 constituyeron una zona de menor riesgo. El comportamiento del accidente ofídico durante los 10 años (2009- 2018) evidenció una muy leve zona de éxito en las semanas 26, 28 y 41, con poca probabilidad de que el evento se reflejara en una zona de seguridad durante ese periodo. Es evidente que las semanas 4, 16, 21, 32, 46 y 49 fueron las de mayor notificación durante todo el periodo (figura 5B).

En cuanto al sexo de los afectados, los accidentes se presentaron en una relación de dos hombres por cada mujer y, del total de los casos, seis (0,36 %) afectaron a mujeres gestantes. El 48,09 % de los casos se presentó en la población joven y adulta entre los 15 y 44 años, clasificada como población económicamente activa. No hubo una diferencia significativa en los casos en cuanto a las etnias (indígenas, negros, mulatos o afrodescendientes) y el mayor número de casos (69,57 %) se registró en población que no se clasificaba en ninguna de estas. La mayoría de las personas afectadas procedían del área rural dispersa (78,40 %) y pertenecían al régimen subsidiado de salud (81,91). El 59,35 % de los casos sucedió cuando las víctimas realizaban labores agrícolas y, al sumar otras actividades laborales menos frecuentes, se puede decir que el 69 % se catalogaría como accidentes de trabajo (cuadro 1). Cabe destacar que, en el medio rural disperso, se registró el 78,40 % de los accidentes y, en los centros poblados y los suburbios, ocurrió el 21,59 %. El número de accidentes en las poblaciones evidencia una presencia importante de serpientes en el medio urbano y suburbano.

Había evidencia de mordedura (marcas de colmillos o dientes) en el 79,73 % de los casos, siendo las extremidades superiores e inferiores las más afectadas (96,73 %). En el 72,60 % de los casos se identificó la serpiente causante, principalmente porque se las capturó o recolectó (42,71 %), y se las describió, consignando su procedencia y el cuadro clínico que presentaba el paciente (cuadro 2).

**Cuadro 1:** Características sociodemográficas del accidente ofídico según registros de Sivigila, 2009-2018

Variable	Característica	n	(%)
Sexo	Masculino	1.124	68,00
	Femenino	523	31,64*
Rango de edad (años)	0-14	295	17,85
	15-29	430	26,01
	30-44	365	22,08
	45-59	340	20,57
	60-74	182	11,01
	75-89	39	2,36
	90 en adelante	2	0,12
Pertenencia étnica	Indígena	252	15,25
	Negro, mulato, afrocolombiano o afrodescendiente	251	15,18
	Ninguna de los anteriores	1.150	69,57
Área de procedencia	Rural disperso	1.296	78,40
	Cabecera	188	11,37
	Centro poblado	169	10,22
Tipo de régimen en salud	Subsidiado	1.354	81,91
	No asegurado	184	11,13
	Contributivo	75	4,54
	Indeterminado	40	2,42
Condición de	Desplazado	11	0,67
desplazamiento	No desplazado	853	51,60
	No determinado	789	47,73
Actividad en el momento	Actividad agrícola	981	59,35
del accidente	Caminar por senderos abiertos o trocha	225	13,61
	Oficios domésticos	169	10,22
	Recreación	98	5,93
	Actividad acuática	21	1,27
	Recolección de desechos	10	0,60
	Otro	149	9,01

* Incluye 6 mujeres gestantes (0,36 %)

### Procedencia

**Figura 6:**
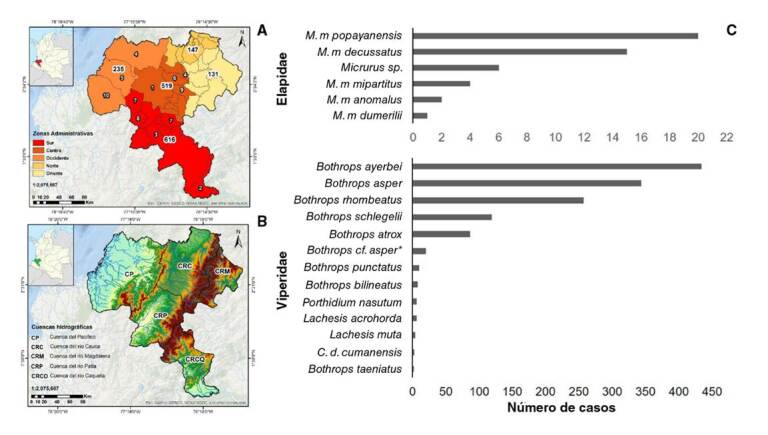
A. Densidad de casos por zonas administrativas del departamento del Cauca (2009-2018). Los números del 1 al 10 corresponden al lugar que ocupa cada municipio con respecto a los 10 con mayor número de casos de ofidismo (cuadro 3). *Los cinco casos faltantes corresponden a municipios desconocidos. B. Cuencas hidrográficas del departamento del Cauca. C. Especies de serpientes causales de accidente ofídico en el departamento del Cauca en el período 2009-2018. *Bothrops de la cuenca del río Magdalena

El departamento se divide en cinco zonas administrativas o provincias (figura 6A) con diversas cuencas hidrográficas. En la zona sur, se registró el mayor número de casos de accidente ofídico (37,32 %), seguida por las zonas centro (31,33 %), occidente (13,97 %), norte (8,71 %) y oriente (8,65 %) (figura 6B). El departamento del Cauca se halla dividido en 42 municipios y en todos ellos hay serpientes de interés clínico. En orden decreciente, los 11 municipios con mayor número de accidentes ofídicos fueron: El Tambo (7,26 %), Piamonte (7,08 %), Bolívar (5,26 %), López de Micay y Piendamó (5,44 % cada uno), Timbiquí (5,20 %), Cajibío (5,08 %), Argelia y La Vega (4,42 % cada uno), Balboa (3,69 %), Popayán (3,63 %) y Guapi (3,57 %) (cuadro 3).

### Zoogeografía de las serpientes en el Cauca

Para comprender la diversidad de la fauna ofídica de este departamento, se presenta su distribución en cinco cuencas hidrográficas: la cuenca del Pacífico, la cuenca del río Cauca, la del río Magdalena, la del río Caquetá y la del río Patía (figura 6B). Las cuencas con mayor diversidad de especies son las del río Caquetá y las del Pacífico, probablemente por la menor intervención antrópica, su variada vegetación, la topografía y el clima.

**Cuadro 2:** Características relacionadas con el agente causal del accidente ofídico y atención inicial recibida por el paciente según los registros del Sivigila en el departamento del Cauca, 2009-2018

Variable	Característica	n	(%)
Huellas de colmillos	Sí	1.318	79,73
	No	333	20,15
	No reporta	2	0,12
Serpiente capturada	Sí	706	42,71
	No	945	57,17
	No reporta	2	0,12
Serpiente identificada	Sí	1.200	72,60
	No	386	23,35
	No reporta	67	4,05
Localización de la	Cabeza (cara)	15	0,91
mordedura	Miembros superiores	842	50,94
	Miembros inferiores	712	43,07
	Dedos de mano	32	1,94
	Dedos de pie y de mano	13	0,79
	Tórax anterior	20	1,21
	Abdomen	5	0,30
	Espalda	2	0,12
	Cuello	2	0,12
	Genitales	1	0,06
	Glúteos	4	0,24
	No reporta	5	0,30
Tipo de atención	Otros	639	38,66
	Torniquete	507	30,67
	Inmovilización del miembro	196	11,86
	Inmovilización del enfermo	85	5,14
	Incisión	74	4,48
	Sangría	54	3,27
	Punción	49	2,96
	Succión mecánica	29	1,75
	Succión bucal	17	1,03
	No determinado	3	0,18

**Cuadro 3:** Número de casos de ofidismo por municipio atendidos en el período de 2009 a 2018

Zona	Municipio	n	Posición
Sur	Piamonte	117	2
	Bolívar	87	3
	Argelia	73	7
	La Vega	73	7
	Balboa	61	8
	Mercaderes	53	
	Patía	53	
	Sucre	40	
	Almaguer	24	
	San Sebastián	15	
	Santa Rosa	12	
	Florencia	8	
Centro	El Tambo	120	1
	Piendamó	90	4
	Cajibío	84	6
	Popayán	60	9
	Morales	54	
	La Sierra	39	
	Rosas	38	
	Timbío	27	
	Sotará	7	
Occidente	López de Micay	90	4
	Timbiquí	86	5
	Guapi	59	10
Norte	Santander de Quilichao	44	
	Corinto	24	
	Suárez	21	
	Buenos Aires	20	
	Caloto	18	
	Miranda	10	
	Guachené	3	
	Puerto Tejada	3	
	Padilla	2	
	Villa Rica	2	
Oriente	Caldono	41	
	Toribío	31	
	Páez	17	
	Inzá	13	
	Totoró	11	
	Jambaló	9	
	Silvia	5	
	Puracé	4	
	Municipio desconocido	5	
TOTAL		1.653	

La gran mayoría de los géneros de colúbridos, dipsádidos opistoglifos, elápidos y vipéridos en Colombia, está representada en el departamento del Cauca (cuadro 4). Las especies con mayor importancia clínica en el departamento son: *Bothriechis schlegelii*, *Bothrops asper*, *B. atrox*, *B. ayerbei*, *B. rhombeatus*, *Chironius monticola*, *Clelia equatoriana*, *Erythrolamprus bizona*, *Spilotes pullatus*, *Micrurus mipartitus*, *Crotalus durissus, Lachesis acrochorda* y *L. muta* (figuras 1, 2, 3 y 4) ^5^^,^^8^^,^^12^^,^^37^.

**Cuadro 4:** Familias, géneros y especies de colúbridos, dipsádidos, vipéridos y elápidos asociados con el accidente ofídico, o potenciales agentes en el Cauca, con los nombres comunes y la distribución por cuencas hidrográficas

Familia	Género	Especie/subespecie	Nombre común	Cuenca hidrográfica
	*Atractus*	*A. multicinctus*	Falsa rabo de ají	CP
	*Chironius*	*C. monticola*	Guache, jueteadora	CRC
	*Clelia*	*C. clelia*	Chonta	CRC
		*C. equatoriana*	Cazadora negra	CP, CRP
	*Drymarchon*	*D. melanurus*	Cazadora	CP, CRP, CRM
	*Drymobius*	*D. rhombifer*	Falsa equis	CP
	*Erythrolamprus*	*E. aesculapii*	Mataganado	CRCQ
		*E. bizona*	Mataganado	CRC, CRP, CRM
		*E. epinephelus*	Falsa coral	CRC, CRM, CRCQ
		*E. guentheri*	Mataganado negra	CRCQ
		*E. mimus*	Falsa coral	CP
Colubridae		*E. pseudocorallus*	Mataganado	CRM
	*Helicops*	*H. angulatus*	Pescadora	CRCQ
		*H. pastazae*	Pescadora	CRCQ
		*H. polylepis*	Pescadora	CRCQ
	*Imantodes*	*I. cenchoa*	Bejuquilla	CP, CRC
	*Lampropeltis*	*L. micropholis*	Mataganado	CP, CRC, CRP, CRM
	*Oxybelis*	*O. aeneus*	Bejuquilla	CRCQ, CP
		*O. brevirostris*	Bejuquilla	CP
	*Oxyrhopus*	*O. leucomelas*	Falsa coral	CRCQ, CRM
		*O. petolarius*	Falsa coral	CP, CRC
	*Philodryas*	*P. olfersii*	Cazadora	CRCQ
	*Rhinobothryum*	*R. bovallii*	Falsa coral	CP, CRM
	*Spilotes*	*S. pullatus*	Coclí, toche voladora	CRC, CRM, CRP, CP
	*Thamnodynastes*	*T. pallidus*	Cazadora	CRCQ
	*Xenodon*	*X. rabdocephalus*	Equis sapa	CRM
Dipsadidae	*Dipsas*	*D. catesbyi*	Dormilona	CRCQ
		*D. sanctijoannis*	Dormilona	CP, CRC, CRM
	*Sibon*	*S. ayerbeorum*	Caracolera	CP
		*S. nebulatus*	Caracolera gris	CP, CRC
	*Bothriechis*	*B. schlegelii*	Cabeza de candado - Yaruma	CRC, CP, CRM
	*Bothrocophias*	*B. colombianus*	Equis colorada	CP
		*B. hyoprorus*	Equis sapa	CRCQ
		*B. microphthalmus*	Mapaná - talla - taya equis	CRCQ
		*B. myersi*	Equis rojiza	CP
	*Bothrops*	*B. asper*	Equis negra - terciopelo	CP
		*B. cf. asper*	Equis del Magdalena	CRM
		*B. atrox*	Cuatro narices	CRCQ
		*B. ayerbei*	Equis patiana - Cacica	CRP
Viperidae		*B. bilineatus*	Lora	CRCQ
		*B. brazili*	Rabo de ratón	CRCQ
		*B. pulcher*	Loro mashako	CRCQ
		*B. punctatus*	Rabo de chucha - orito	CP
		*B. rhombeatus*	Equis gata - equis pelo de gato	CRC
		*B. taeniatus*	Estrella	CRCQ
	*Crotalus*	*C. durissus*	Cascabel	CRM
	*Lachesis*	*L. acrochorda*	Verrugosa	CP
		*L. muta*	Guascama - rieca - surucucú	CRCQ
	*Porthidium*	*P. nasutum*	Hilván - patoquilla	CP
	*Hydrophis*	*H. platurus*	Serpiente marina	Océano Pacífico
	*Micrurus*	*M. narduccii melanotus*	Coral negra esbelta	CRCQ
		*M. ancoralis ancoralis*	Coral ancla ecuatoriana	CP
		*jani*	Coral ancla de Jan	CP
		*M. clarki*	Coral de Clark	CP
		*M. dumerilii antioquiensis*	Coral antioqueña	CRC
		*carinicauda*	Coral capuchina	CRM
		*transandinus*	Coral transandina	CP
Elapidae		*M. mipartitus anomalus*	Rabo de ají anómala	CRM
		*decussatus*	Rabo de ají andina	CRC
		*mipartitus*	Rabo de ají del Pacífico	CP
		*popayanensis*	Rabo de ají payanesa	CRP
		*M. multifasciatus*	Coral panameña	CP
		*M. multiscutatus*	Coral caucana	CP
		*M. obscurus*	Coral amazónica	CRCQ
		*M. oligoanellatus*	Coral de Tambito	CP
		*M. ornatissimus*	Coral ornamentada	CRCQ
		*M. surinamensis*	Coral acuática	CRCQ

CP: cuenca del Pacífico; CRC: cuenca del río Cauca; CRCQ: cuenca del río Caquetá; CRM: cuenca del río Magdalena; CRP: cuenca del río Patía. cf.: por confirmar

**Cuadro 5:** Géneros de serpientes causantes de accidente ofídico en el departamento del Cauca y casos reportados de departamentos vecinos durante el período de 2009 a 2018. Desc.: desconocido

Departamentos	*Bothrops, sensu lato*	*Micrurus* spp.	*Lachesis* spp.	*Crotalus durissus*	Colubridae	Desc.	Total
Cauca	1.280	48	10	3	35	277	1.653
Putumayo	2	2	-	-	-	2	6
Caquetá	-	-	1	-	-	2	3
Nariño	2	-	-	-	-	1	3
Valle del Cauca	2	-	-	-	-	1	3
Huila	-	-	-	-	-	1	1
Tolima	-	1	-	-	-	-	1
Indeterminado	4	-	-	-	-	-	4
Total	1.290	51	11	3	35	284	1.674

En el cuadro 4, se agrupan bajo la denominación *Bothrops, sensu lato* algunos géneros de víboras cuyos venenos presentan un cuadro clínico similar, denominado síndrome botrópico (*Bothrops*, *Bothriechis*, *Porthidium*), el cual representa el 77,43 % de los casos. El género *Micrurus* (corales verdaderas) ocasionó el 2,9 %, *Lachesis* el 0,6 %, la familia Colubridae el 2,11 %, el género *Crotalus* apenas registró 0,18 % y en el 16,75 % de los casos no se logró identificar el agente causal. Cabe señalar que en el departamento del Cauca se atendieron 17 casos ocurridos en otros departamentos, en su mayoría provenientes del departamento del Putumayo (cuadro 5).

Entre las serpientes de mayor interés clínico involucradas en los accidentes ofídicos en el Cauca, se identificaron 20 especies venenosas pertenecientes a las familias Viperidae y Elapidae, así como varias de la familia Colubridae, algunas de ellas con dentición opistoglifa, pero cuyos venenos no constituyen un peligro grave para el ser humano (figura 6C). Se encontró que cuatro especies ocasionaron el mayor número de accidentes: *Bothrops ayerbei*, conocida como “equis patiana” o “cacica”, con el 32 % de las mordeduras cuyo agente causal fue identificado, seguida por *B. asper*, llamada “terciopelo” o “equis negra”, con el 24,4 %, *B. rhombeatus*, la denominada “equis gata” o “pelo de gato”, con un 18,1 %, y *Bothriechis schlegelii*, en el cuarto lugar, con el 8,4 %. En el 16,4 % de los accidentes no se logró identificar el agente causal y tampoco se sospechó de ningún género, pues los afectados no presentaban un síndrome definido, lo que podía corresponder a mordeduras en seco o “de aviso” de *Bothrops* spp. o de serpientes no venenosas. Vale anotar que en este estudio no se reportó o reconoció ningún accidente causado por el género *Bothrocophias* (figura 6C), a diferencia de lo registrado en estudios previos ^20^^,^^22^^,^^38^.

### Manejo del accidente ofídico

En el cuadro 2 se presenta el tipo de atención inicial que recibieron los pacientes. Se logró determinar que el 30,67 % de las prácticas no médicas a las que recurren los pacientes antes de ser atendidos en un centro hospitalario, corresponde al uso de torniquetes y, el 38,66 %, correspondió a diferentes prácticas a cargo de curanderos, como aguas, hierbas, alcohol, gasolina, agua fría, tabaco, orina, yodopovidona, limón, compresas, incisiones, emplasto, petróleo, imán, lavado o limpieza de la herida y masajes, las que generalmente empeoran el estado de salud de la persona o influyen en el retraso de la consulta en el nivel 1 de atención médica.

**Cuadro 6:** Gravedad del accidente ofídico y aplicación u omisión de antiveneno en el departamento del Cauca, 2009-2018****

Variable	Característica	n	%	Antiveneno, sí	%	Antiveneno, no	%
Grado del	Sin envenenamiento	152	9,20	-	-	580	61,05
accidente	Leve	841	50,88	261	37,13	178	18,74
	Moderado	480	29,04	302	42,96	36	3,79
	Grave	175	10,59	139	19,77	152	16,00
	No reporta	5	0,30	1	0,14	4	0,42
Total		1.653		703		950	

En el cuadro 6 se muestra el manejo de los casos sin envenenamiento, envenenamiento leve, moderado y grave, señalando el número, el porcentaje, y el uso o la omisión de antiveneno o suero antiofídico durante el periodo de estudio. El antiveneno se utilizó en 703 casos (42,53 %) así: polivalente en 676 (96,16 %), monovalente (que todavía se producía en la primera década del presente siglo) en 17 casos (2,41 %) y el anticoral en 10 casos (1,42%). Cabe señalar que se diferenciaron los tres tipos de suero antiofídico (polivalente: antibotrópico, antilachésico, anticrotálico; monovalente, y anticoral), porque así se consigna la información en las fichas de notificación INS: 100 en las versiones de 2009 a 2018.

Con base en los tipos de accidentes ofídicos y el análisis de las variables, se proponen 12 recomendaciones generales para una correcta atención del paciente y un adecuado registro del accidente ante las instancias pertinentes. Es muy importante considerar que cada caso de ofidismo es diferente debido a la variabilidad intraespecífica e interespecífica de las serpientes y sus venenos a nivel geográfico, ontológico, sexual y dietario, entre otros, así como a las condiciones y comorbilidades propias de cada paciente. Así:

1. Establecer si realmente el paciente presenta mordedura de serpiente

2. Determinar si hay envenenamiento o no lo hay (mordedura en seco)

3. Establecer el tiempo transcurrido desde que ocurrió el accidente

4. Precisar si se hizo algún procedimiento inicial (médico o de otro tipo) al paciente que pudiera contrarrestar el tratamiento o agravar su estado

5. Establecer la procedencia geográfica y verificar las especies de serpientes comunes en el área (pueden emplearse los cuadros y figuras del presente estudio como respaldo)

6. Identificar el agente causal (con base en la serpiente capturada, el registro fotográfico o los signos y síntomas asociados). Esta información, así como la de los puntos 3, 4 y 5, permitiría anticipar algunas manifestaciones y complicaciones a partir del conocimiento del tipo de veneno de la especie y su mecanismo de acción (proteolíticos, coagulantes, hemolíticos, necrotóxicos, neurotóxicos, vasculotóxicos, hepatotóxicos, nefrotóxicos o miotóxicos, entre otros).

7. Diagnosticar el tipo de accidente ofídico (botrópico, elapídico (micrúrico o pelámico (*Hydrophis platurus*), colúbrido, lachésico, crotálico o boideo)

8. Establecer el grado de envenenamiento

9. Diagnosticar complicaciones y enfermedades previas del paciente

10. Ordenar exámenes paraclínicos para correlacionarlos con la clínica, y determinar el tipo y las dosis adecuadas de suero antiofídico

11. Coordinar el equipo médico y paramédico interdisciplinario para el manejo integral del paciente.

12. Hacer un correcto y completo diligenciamiento de la ficha de notificación INS:100 y, si esta ya se ha tramitado, verificar los datos.

**Cuadro 7:** Comparación de los tipos de ofidismo observados en diferentes estudios realizados en el departamento del Cauca

Período	Botrópico	Colúbrico	Micrúrico	Lachésico	Crotálico	Desconocido	n
1972-1975	35	10	3	-	-	-	48
1993-1997	63	2	-	1	-	-	66
2000-2008	333	30	2	-	3	14	382
2009-2018	1.280	35	48	10	3	277	1.653

## Discusión

### Tipos de accidentes ofídicos

El comportamiento del accidente ofídico en el departamento del Cauca evidenció cambios con respecto a estudios anteriores. En el primer estudio sobre ofidismo entre 1977 y 1979, de Ayerbe, *et al.,* solo se registraron tres tipos de accidentes: botrópico (72,9 %), colúbrico (20,8 %) y micrúrico (6,25%) (7,20). En el 2000, en otro estudio de Ayerbe, se registraron también tres tipos de accidentes -botrópico (95,5 %), colúbrico (3 %) y lachésico (1,5)- con un aumento de casos de ofidismo botrópico y el registro de un nuevo tipo de accidente ^38^. A comienzos del siglo XXI, Ayerbe, *et al.*, registraron envenenamientos botrópicos (87,2 %), colúbricos (7,85 %), crotálicos (0,78 %) y micrúricos (0,52 %), así como un porcentaje no identificado (3,66 %) ^5^. En el presente estudio. se presentaron cinco tipos de ofidismo: botrópico (77,43 %), micrúrico (2,9 %), colúbrico (2,12 %), lachésico (0,60 %) y crotálico (0,18 %), y un porcentaje no identificado (16,76 %), lo cual evidenció una tendencia cambiante de la enfermedad en el departamento (cuadro 7), que se relacionaría con el aumento de la población y del número de registros, ya que desde el 2004 es de notificación obligatoria al Sivigila, aunque persiste un elevado subreporte.

El ofidismo botrópico sigue ocupando el primer lugar con porcentajes que han oscilado entre 72,9 y 95,3 % (ẋ=83,32 %), semejante a lo reportado en otras partes de Colombia y Latinoamérica ^2^^,^^14^^,^^23^^,^^30^^,^^39^^,^^40^. Se aclara que en estudios más recientes, del 2018 ^2^, el 2019 ^14^ y el 2020 ^9^ en departamentos geográficamente cercanos, se ha registrado una tendencia al aumento del número de casos y el tipo de ofidismo, pero con propensión a la disminución en los porcentajes; así, se aprecia que el ofidismo colúbrico, que ocupó el segundo lugar, bajó del 20,8 % al 2 % (90,38 %) y el micrúrico, en el tercer lugar, bajó del 6,25 % al 2,9 % (50,88 %).

Por otra parte, el ofidismo no identificado o desconocido, que no aparecía en los dos primeros estudios, se incrementó cuatro veces más, del 3,66 al 16,35 % (cuadro 7), variaciones que se explicarían por el hecho de que el número de casos (n) en las dos primeras series era muy reducido, ya que no se reportaban a los centros hospitalarios, aunque en los casos que llegaban al hospital el agente estaba bien identificado. En las dos últimas series solo se identificaron los casos remitidos con cuadros clínicos bien definidos, pero cuando eran mordeduras sin envenenamiento, era difícil establecer si se trataba de mordeduras “en seco” o producidas por colúbridos.

### Frecuencia e incidencia de los accidentes ofídicos

Los accidentes ofídicos en el departamento del Cauca evidenciaron cambios en la casuística del evento comparados con los estudios iniciales de 1977, 1979, 2000, 2001 y 2009 ^7^^,^^20^^-^^22^^,^^38^. En dichos estudios, se registró una mayor incidencia en los municipios con acceso directo a la vía Panamericana, así como un menor número de casos promedio por año. En el presente estudio, esa tendencia cambió, lo cual puede atribuirse a una mejor disponibilidad de medios de transporte terrestre, fluvial y aéreo, además de la disminución en el subregistro de casos a raíz de la obligatoriedad de notificación del accidente ofídico al Sivigila desde octubre del 2004 (Circular 092 del Ministerio de Salud de Colombia), aunque la notificación empezó a mejorar desde el 2007, lo que ha permitido tener una mejor aproximación al número de registros del ofidismo en el departamento. Asimismo, en los estudios iniciales prácticamente los únicos casos que se conocían eran los que llegaban al Hospital Universitario San José, en donde se implementó un proceso de atención personalizada a las víctimas de ofidismo desde 1980 hasta el 2008, medida que ha demostrado ser efectiva, pues se evidenció un aumento en las estadísticas de atención, pasando de 12 casos entre 1972 y 1975, a 13 y 42 posteriormente, y hasta el actual reporte de 165,3 casos anuales en promedio, lo que equivale a un incremento del 92,91 % que, aunque relacionado parcialmente con el aumento de población en el departamento, evidencia claramente una mayor notificación de los casos.

Las cifras observadas entre el 2009 y el 2018 son equiparables a las reportadas en la casuística de otros departamentos, así como de varias regiones de países sudamericanos con presencia de serpientes de géneros similares a los del departamento del Cauca (Cardoso J. Aspectos clínicos y epidemiológicos del accidente ofídico en Brasil. Memorias del Primer Simposio Colombiano de Toxinología. Medellín: Ecográficas Ltda.; 1998. p. 77-80). ^10^^,^^29^^,^^30^^,^^39^^,^^40^^-^^44^. Específicamente, el departamento del Cauca comparte rasgos biomédicos y epidemiológicos del accidente ofídico con los departamentos del suroccidente colombiano, Valle del Cauca ^43^ y Nariño, registrando este último un mayor número de defunciones asociadas principalmente con la demora en la búsqueda de atención médica por parte de los pacientes ^14^. En cuanto a la incidencia del accidente, Cauca superó la nacional, que osciló entre 7,5 y 10,1 (2009-2017), durante todo el periodo de estudio, por lo que este departamento se convierte en uno de los que más requieren atención de esta enfermedad neotropical desatendida ^16^^-^^18^^,^^45^^-^^52^.

Es importante resaltar que, en todos los años, los casos tendieron a elevarse en ciertos periodos epidemiológicos, como se evidencia en la tendencia anual y el canal endémico (figuras 5 y 6A). Esto ha permitido revelar una tendencia al aumento de casos en las semanas 4, 16, 21, 32, 46 y 49, las de mayor notificación durante todo el periodo, destacándose el año 2016 como el de mayor número de casos y el 2017 como el año en el que se inició un descenso en las notificaciones, lo que coincide con algunos periodos en el canal endémico del departamento de Magdalena entre 2009 y 2013. Se evidenció una tendencia similar frente a los datos nacionales, ya que en el 2016 la incidencia de accidentes ofídicos en el país fue de 10 casos por cada 100.000 habitantes, siendo Antioquia el departamento con el mayor número de casos y, Nariño, el que registró un descenso de la notificación en los últimos años ^14^^,^^23^.

La mortalidad por ofidismo descendió paulatinamente de 6,2 % en 1979 a cero en el 2000 y luego se incrementó levemente a 1,83 % en el 2009 ^5^^,^^7^^,^^38^. Actualmente, se encuentra en 0,3 %, por debajo de la tendencia nacional y la de los departamentos vecinos, como Nariño, donde se registró un 2,43 % ^14^. En diferentes regiones de Colombia, se han reportado tasas de mortalidad entre 1,8 y 9 % ^2^^,^^29^^,^^53^. La disminución en la tasa de mortalidad en el Cauca probablemente se asocia con la consulta rápida, la buena disponibilidad, la calidad y la correcta aplicación del antiveneno, la asesoría rápida y efectiva, una adecuada identificación de los tipos de ofidismo y un mejor conocimiento de la fisiopatología de los venenos. Es posible que la mortalidad no se reduzca nuevamente a cero, pues ha llegado personal médico de otras regiones del país, que desconoce el comportamiento de esta enfermedad en el departamento, y puede haber renuencia, incapacidad o dificultad para remitir al paciente al nivel 3 de atención medica oportunamente. Por esta razón, es importante integrar la cátedra de Toxinología en la enseñanza de las profesiones relacionadas con la atención y el estudio del accidente ofídico, capacitar al personal encargado de diligenciar las fichas de notificaciones INS: 100 para evitar la tergiversación de la información, y establecer un comité de vigilancia epidemiológica interinstitucional que revise periódicamente la casuística del accidente ofídico para, así, mejorar su tratamiento, prevención, y epidemiología.

En cuanto a las variables de sexo, rango de edad, pertenencia étnica, área de procedencia, tipo de régimen en salud, condición de desplazamiento, actividad en el momento del accidente, huellas de colmillos, serpiente capturada e identificada y localización de la mordedura, estas son coherentes con las tendencias generales de los estudios municipales y departamentales, así como con los informes a nivel nacional ^2^^,^^3^^,^^13^^,^^14^^,^^16^^,^^17^^,^^21^^,^^23^^,^^26^^,^^38^^,^^41^^-^^43^^,^^46^^-^^52^^,^^54^^-^^56^, en los que también se reporta que las personas más afectadas son quienes se dedican a la agricultura o a actividades afines en el campo, actividades tradicionalmente desarrolladas por hombres. Por ello, la mayoría de los casos se registra en hombres en edad económicamente productiva, es decir, en los rangos de edad entre los 15 y los 29 años y entre los 30 y los 44 años ^52^. Asimismo, en las poblaciones afrocolombianas e indígenas se registra un mayor riesgo de sufrir accidentes ofídicos, probablemente porque habitan predominantemente viviendas rurales en condiciones ecológicas propicias para la proliferación de serpientes y se dedican a labores de campo sin utilizar elementos de protección por razones culturales o económicas ^52^^,^^55^.

También, es importante señalar que se registró un número considerable de casos en que no fue posible identificar el tipo de accidente (16,76 %), lo que concuerda con el número de casos en que no se pudo identificar la serpiente (23,35 %). Esto evidencia la falta de conocimientos taxonómicos y geográficos en el personal de salud y en la población en general, para determinar las características específicas de cada género ^14^^,^^20^^,^^23^.

### Procedencia y zoogeografía de las serpientes en el Cauca

Todos los municipios notificaron al Sivigila casos de accidente ofídico, al igual que sucedió en el departamento de Sucre ^43^, y por encima de Nariño (78,13 %) ^14^ y Magdalena (96,5 %) ^23^. Estos porcentajes están estrechamente ligados con el número de municipios de cada departamento. Los municipios más afectados por el accidente ofídico fueron aquellos localizados en las zonas sur, centro y occidente, lo que concuerda con los resultados expuestos por Cuéllar, *et al.*, y Sevilla-Sánchez, *et al.*^14^^,^^23^. Estos municipios se caracterizan por la presencia de ecosistemas aptos para las serpientes por su régimen de lluvias, la existencia de zonas de bosque pluvial premontano, húmedo tropical y seco tropical, y el hecho de abarcar desde el nivel del mar hasta los 2.000 metros de altura. Además, el comportamiento del accidente ofídico coincide con el de estudios retrospectivos similares y los informes anuales de eventos entregados por el Instituto Nacional de Salud ^14^^,^^16^^-^^18^^,^^23^^,^^29^^,^^40^^,^^42^^-^^44^^,^^46^^-^^51^.

Geográficamente, Colombia está situada en el punto de unión entre Centroamérica y Sudamérica. Desde el punto de vista orográfico, en el país se trifurca la cordillera de los Andes, lo cual genera una gran variedad de ecosistemas y, a su vez, explica la gran biodiversidad que posee. En este contexto, en el departamento del Cauca se originan las tres ramas de los Andes y cuatro de los ríos más importantes del país, lo cual lo convierte en una de las regiones más biodiversas de Colombia y quizá una de las más ricas del planeta en especies vivientes. Según Lynch ^13^, las serpientes ocupan la mayoría de los hábitats, con una gran diversidad en tierras bajas y cálidas, diversidad que disminuye de manera importante cuando aumenta la altitud, lo que se ajusta con el perfil epidemiológico de las mordeduras de serpiente en el departamento del Cauca, donde los casos se presentan principalmente en las zonas sur, central y occidental, correspondientes a las regiones naturales del Pacífico y andina de Colombia, catalogadas como las más biodiversas ^24^^,^^32^.

Asimismo, las zonas con el mayor número de casos coinciden con las áreas de distribución de las especies a las cuales se les atribuye el mayor número de casos de ofidismo en el Cauca, en otros departamentos y a nivel nacional y latinoamericano, como sucede con el género *Bothrops*^2^^,^^4^^,^^9^^,^^14^^,^^16^^,^^18^^,^^19^^,^^23^^,^^43^^,^^44^^,^^46^^,^^47^^,^^51^.

*Bothrops ayerbei*, causante de la mayoría de los accidentes, se distribuye en la cuenca alta del río Patía, en la zona sur del Cauca, y habita en el bosque premontano y subtropical entre los 400 y los 1.800 msnm, desde la margen sur del divorcio de aguas de los ríos Cauca y Patía en los municipios de Popayán, Timbío y el sudeste de El Tambo, hasta la cuenca del río Patía, incluido el norte de Nariño en Taminango y Colón ^37^. Asimismo, se infiere la presencia de esta especie en la región andina del Ecuador por las similitudes morfológicas y "venómicas" (sic) de los ejemplares estudiados en el sudeste de Colombia y el noreste del Ecuador ^56^.

*Bothrops asper,* por su parte, se encuentra en el bosque húmedo tropical premontano y subtropical entre el nivel del mar y los 1.800 msnm en la zona occidental propia del Chocó biogeográfico del departamento del Cauca, en los municipios de Guapi, Timbiquí y López de Micay, y al oeste de los municipios de Argelia y El Tambo, teniendo como barrera natural la cordillera Occidental. En esta especie, se ha evidenciado variabilidad en la composición proteica de su veneno a nivel intraespecífico a lo largo de su rango de distribución, incluidas las especies del departamento del Cauca ^8^^,^^37^^,^^57^.

*Bothrops rhombeatus* se encuentra en el bosque subtropical desde los 1.000 msnm hasta el bosque montano a 2.600 msnm en El Tambo (vertiente noreste de la cordillera Occidental asociada con la cuenca del río Cauca) y desde el altiplano de Popayán tomando como punto de partida de sur a norte el divorcio de aguas de los ríos Patía y Cauca en los municipios de Popayán, Timbío y El Tambo hacia el Valle del Cauca, el eje cafetero y Antioquia ^9^^,^^37^, lo que coincide con los municipios que reportaron el mayor número de casos de ofidismo en el departamento.

*Bothriechis schlegelii*, que ocupa un lugar importante como agente causal de mordeduras en este departamento, se localiza en la cuenca del Pacífico y de los ríos Cauca y Magdalena desde el nivel del mar hasta los 2.800 msnm, en el bosque montano. Es de hábitos arbóreos y, aunque su veneno es de baja toxicidad, en ocasiones produce mordeduras en cuello y cara, lo que genera situaciones peligrosas (figura 1A) ^5^^,^^10^^,^^13^^,^^33^. Muchos recolectores de café son mordidos por esta especie.

Por otra parte, las especies propias del género *Micrurus* fueron las responsables del 2,9 % de los accidentes ofídicos en el Cauca, especialmente dos subespecies: *Micrurus mipartitus decussatus* y *M. mipartitus popayanensis,* con 35 de los 48 casos. Estas subespecies presentan una distribución asociada con las cuencas del río Cauca y del Patía, respectivamente, siendo propias de las zonas centro y sur del departamento, donde se registra el mayor número de casos ^58^. Ambas subespecies se han registrado en los municipios de Bolívar, La Vega, Rosas, Santander de Quilichao y Popayán, principalmente en las cordilleras Occidental y Central ^58^, distribuidas desde los ecosistemas secos o desérticos hasta los bosques húmedos e hiperpluviales con zonas boscosas, semiboscosas, parches de bosques, bosques húmedos pluvial tropical y premontano y, en ocasiones, cerca de asentamientos humanos, donde hay minería y agricultura, lo cual explica los accidentes que se presentan ^59^.

### Manejo del accidente

En cuanto a las prácticas no médicas, se encontró que la más utilizada durante la atención inicial es la colocación del torniquete, seguida de otras prácticas no identificadas, lo cual coincide con lo registrado en otros estudios ^14^^,^^23^^,^^43^^,^^60^. Según los resultados de Otero, *et al.*^60^, el uso del torniquete no empeoró la intensidad del envenenamiento botrópico, hecho que se puede explicar porque en esos casos no transcurrió mucho tiempo entre su colocación y el inicio del manejo indicado en los protocolos. Sin embargo, basados en la lógica y la experiencia, otros autores afirman que el uso del torniquete, la crioterapia y la succión, deben evitarse como medidas preventivas de primeros auxilios, pues tienen riesgos, facilitan las complicaciones y afectan el pronóstico del paciente, retrasando el tiempo de aplicación de los antídotos y potenciando las secuelas ^60^.

La mayoría de los envenenamientos se trataron con los antivenenos polivalentes disponibles en Colombia, los cuales son capaces de neutralizar el veneno de varios géneros de serpientes de la misma familia (*Bothrops*, *Crotalus* y *Lachesis*) asociados con el accidente ofídico botrópico ^61^. Después de la mordedura, son decisivas las dos primeras horas para atenuar los efectos del veneno; sin embargo, muchos pacientes reciben el antiveneno después de este tiempo, lo que puede deberse a que no se dispone de él o no se sabe cómo aplicarlo, a que no se utiliza aunque se disponga de él por el temor infundado a las complicaciones que pueda generar ^62^, o a que al ser un recurso muy preciado y escaso, la decisión de aplicarlo no es sencilla. También, la distancia entre los sitios de la mayoría de los accidentes y el centro hospitalario más cercano, hace que se pierdan horas cruciales. Pese a ello, en el Cauca suele usarse el antiveneno ajustándose al tratamiento adecuado del paciente intoxicado, así como a los protocolos de manejo establecidos para el accidente ofídico ^1^^,^^26^^,^^55^. No se desconoce, sin embargo, que las complicaciones pueden prevenirse mejorando el cumplimiento y la aplicación del protocolo del Instituto Nacional de Salud ^27^.

El análisis retrospectivo del presente estudio constituye una aproximación actualizada al accidente ofídico en el departamento del Cauca hasta el 2018, lo que lo convierte en uno de los departamentos colombianos en donde los análisis del ofidismo cubren más tiempo, con estudios desde 1977. En el mismo sentido, se puede concluir que las zonas más afectadas son el sur y el centro, donde las cuencas más asociadas con el accidente son las de los ríos Patía y Cauca, siendo los municipios de El Tambo, Piamonte y Bolívar los que presentan el mayor número de casos ocasionados por los géneros *Bothrops* y *Bothriechis*, particularmente las especies *B. ayerbei*, *B. asper*, *B. rhombeatus* y *B. schlegelii*, generalmente en el área rural dispersa y en la población masculina en edades económicamente activas (15 a 29 y 30 a 44 años).

Es fundamental que la Secretaría de Salud Departamental implemente un método de educación continuada para instruir a los médicos que llegan semestralmente a ocupar las plazas rurales, y que se reestablezca la enseñanza de la toxinología en las facultades de medicina, enfermería, fisioterapia y biología para capacitar a los estudiantes en el conocimiento y manejo de las enfermedades causadas por los venenos, toxinas y ponzoñas que a diario afectan a las poblaciones rurales más vulnerables; además, se debe fortalecer la auditoría a las empresas administradoras de planes de salud y las instituciones prestadoras de servicios en materia de verificación del cumplimiento de las actualizaciones que deben brindarse al personal asistencial en torno a la atención del accidente ofídico.
